# Interaction of immune checkpoint PD-1 and chemokine receptor 4 (CXCR4) promotes a malignant phenotype in pancreatic cancer cells

**DOI:** 10.1371/journal.pone.0270832

**Published:** 2022-07-07

**Authors:** Megan M. Harper, Miranda Lin, Michael J. Cavnar, Prakash K. Pandalai, Reema A. Patel, Mei Gao, Joseph Kim

**Affiliations:** 1 Division of Surgical Oncology, University of Kentucky, Lexington, Kentucky, United States of America; 2 Division of Medical Oncology, University of Kentucky, Lexington, Kentucky, United States of America; Institute of Biomedical Sciences, TAIWAN

## Abstract

Despite recent therapeutic advances, pancreatic ductal adenocarcinoma (PDAC) remains a devastating disease with limited therapeutic options. Immune checkpoint inhibitors (ICIs) have demonstrated promising results in many cancers, but thus far have yielded little clinical benefit in PDAC. Based on recent combined targeting of programmed cell death protein-1 (PD-1) and C-X-C chemokine receptor 4 (CXCR4) in patient-derived xenografts (PDXs) and a pilot clinical trial, we sought to elucidate potential interactions between PD-1 and CXCR4. We observed concomitant expression and direct interaction of PD-1 and CXCR4 in PDAC cells. This interaction was disrupted upon CXCR4 antagonism with AMD3100 and led to increased cell surface expression of PD-1. Importantly, CXCR4-mediated PDAC cell migration was also blocked by PD-1 inhibition. Our work provides a possible mechanism by which prior studies have demonstrated that combined CXCR4 and PD-1 inhibition leads to decreased tumor growth. This is the first report investigating PD-1 and CXCR4 interactions in PDAC cells and our results can serve as the basis for further investigation of combined therapeutic targeting of CXCR4 and PD-1.

## Introduction

Immune checkpoint inhibitors (ICIs) have revolutionized therapeutic cancer regimens by activating quiescent cytotoxic immune cells to eradicate tumor cells. Despite impressive tumor regression and long-term survival benefits with these therapies in patients with various advanced cancers, a large number of cancer patients do not benefit from ICIs. In fact, clinical trials have shown that single-agent ICIs are generally ineffective in patients with pancreatic ductal adenocarcinoma (PDAC), the most common form of pancreatic cancer [1, 2]. This lack of clinical efficacy is likely multifactorial. It can be attributed in part to low tumor mutation burden and therefore a low concentration of immunogenic neoantigens that can be recognized by the immune system [3]. Additionally, dense desmoplasia surrounding PDAC tumors may preclude drug infiltration into the tumor [4]. We, along with other groups, have detected tumor-intrinsic PD-1 expression in PDAC, melanoma, and ovarian cancer [5–8]. These reports show that cancer-cell intrinsic PD-1 activates and regulates multiple signaling pathways to promote tumor growth and escape pathways, thus mitigating treatment response to single-agent ICIs. Furthermore, other reports have shown that antigen presenting cells (APCs) and tumor-intrinsic PD-1 promote immune tolerance to cancer cells [5]. However, much still remains to be learned regarding tumor-intrinsic PD-1 expression and treatment response to ICIs.

In prior reports, the C-X-C chemokine receptor 4 (CXCR4) antagonist AMD3100 has been combined with anti-PD-1 or anti-PD-L1 antibodies in PDAC models to enhance ICI efficacy [9, 10]. In 2013, Feig and colleagues theorized that fibroblast cells in the tumor microenvironment (TME) produced the C-X-C motif chemokine 12 (CXCL12), the ligand of CXCR4, which antagonizes and blocks the immune response from killing PDAC cells [9]. They discovered that CXCR4 inhibition with AMD3100 attenuated CXCL12 release from cancer-associated fibroblasts (CAFs), and concomitant treatment with an anti-PD-L1 antibody reactivated tumor immune recognition and eradicated PDAC in a murine model [9]. Overall, Feig *et al*., concluded that inhibition of CAFs with AMD3100 reduced tumor-stromal interactions, increased tumor T-cell infiltration, and enhanced the therapeutic efficacy of ICIs. More recent work by Seo *et al*. supports this mechanism revealing that combined blockade of PD-1 and CXCR4 results in migration of CD8+ T-cells to the TME and enhances tumor cytotoxicity in an *ex vivo* human PDAC slice culture system [10]. However, both studies by Seo and Feig omitted examination of CXCR4 and immune checkpoint expression in PDAC cells and the effects of AMD3100 on these cells.

Combined therapeutic targeting of CXCR4 and PD-1 appears promising and is under phase 1 and 2 clinical trial evaluation for various cancers (NCT04058145, NCT03628859, NCT04177810, NCT02826486, NCT03168139, and NCT04177810) [11–13]. However, these trials focus wholly on cytotoxicity generated by immune responses. Our objective in this study was to characterize endogenous cancer cell PD-1 and CXCR4 interactions that contribute to the overall cytotoxicity from these combination regimens. While a few studies have evaluated the synergy of anti-PD-1 antibodies with CXCR4 antagonism in PDAC, none has investigated this combination in PDAC independent of immune response. Based on our previous findings demonstrating endogenous PD-1 expression in PDAC cells and ongoing investigations evaluating the efficacy of combining ICIs and CXCR4 antagonism, we sought to further characterize PD-1 and CXCR4 expression in PDAC cells.

## Materials and methods

### Cell culture

The established PDAC cell lines MIAPaCa-2 and PANC-1 and acute lymphoblastic leukemia (ALL) line MOLT-4 were obtained from ATCC. MIAPaCa-2 and PANC-1 cells were cultured in Dulbecco’s Modified Eagle Media (DMEM) (Gibco) supplemented with 10% fetal bovine serum (FBS). RPMI 1640 media (Gibco) supplemented with 10% FBS was used for MOLT-4 cells. All cell lines were maintained at 37°C in a humidified atmosphere at 5% CO_2_. Cell lines were passaged every 3–4 days at 70–80% confluence.

### Patient recruitment and PDAC organoid creation

The study was conducted according to the guidelines of the Declaration of Helsinki. Written informed consent was obtained from PDAC patients undergoing standard-of-care surgery to provide tumor specimens. We obtained Institutional Review Board approval at the University of Kentucky for tissue acquisition, patient-derived organoid (PDO) generation, and subsequent analyses (protocol 48495, approved 1/17/2019). Patient samples were catalogued using the nomenclature “hPT#,” for human PDAC, where hPT# tumor, hPT# PDO, *etc*., were all derived from the same patient. We generated PDOs as previously described [6, 14, 15]. Briefly, PDAC tissues were minced and digested with collagenase II and dispase in AdDF (advanced DMEM/F12 medium supplemented with FBS, Glutamax, HEPES, and primocin) wash medium at 37°C with constant agitation for 30–60 min. The digestion was stopped and the samples were centrifuged (200×g) at 4°C for 5 min. The supernatant was removed and centrifuged again. The pellets were combined and washed twice with AdDF wash medium followed by centrifugation (200×g) at 4°C for 5 min. The cell pellet was resuspended in reduced growth factor basement membrane extract (RGF BME; Trevigen) and cultured in complete PDO medium supplemented with Y27632 (10 μM), generated as previously described [6, 14, 15].

Culture medium was exchanged every 2–3 d and PDOs were passaged every 5–7 d when 70–80% confluent. For passaging, culture medium was removed and PDOs along with RGF BME were collected in cold dispase (1 mg/mL) in AdDF wash media and kept on ice for 10 min. PDOs were then mechanically disrupted with gentle pipetting. PDOs were then centrifuged (200×g) for 5 min at 4°C and the supernatant was carefully removed. The PDO pellet was then resuspended in fresh RGF BME and plated on a 24-well plate in a 1:2 fashion for expansion, or biobanked in AdDF media with 10% FBS and 10% DMSO and stored in liquid nitrogen for later use.

### Western blot assays

We performed western blot assays to determine expression of PD-1 and CXCR4 in PDAC cells and PDOs. The cells were lysed with RIPA lysis buffer (Cell Signaling Technology) supplemented with phosphatase and protease inhibitors (Sigma-Aldrich). Protein concentrations were determined using the Pierce^™^ bicinchoninic acid (BCA) kit (ThermoFisher). Protein samples (20–40 μg) were electrophoresed in 10% SDS polyacrylamide gels and transferred to polyvinylidene difluoride membranes (Bio-Rad). The membranes were blocked with 5% milk and incubated overnight at 4°C with primary antibodies against PD-1 (1:1000, Proteintech, 66220-1-Ig), CXCR4 (1:1000, Proteintech, 60042-1-Ig), or β-actin (1:5000, Sigma-Aldrich) for loading control. Then the blots were incubated for 1 h at room temperature with a corresponding HRP-conjugated secondary antibody (1:5000; Santa Cruz Biotechnology), visualized in ECL solution (SuperSignal West Pico Chemiluminescent Substrate, ThermoFisher) and exposed with an UVP ChemiDoc-It2imager. Blots were quantified using ImageJ (NIH) and results analyzed using GraphPad software.

### Immunofluorescent staining

Immunofluorescence (IF) assays were performed to determine the expression and localization of PD-1 and CXCR4 on primary PDAC tissues, PDAC cells, and PDOs. α-smooth muscle actin (α-SMA) was used to determine localization of cancer-associated fibroblasts (CAFs). Since α-SMA can be expressed in cancer cells, we utilized architectural characterization to differentiate α-SMA+ CAFs from PDAC cells. For staining of MIAPaCa-2 and PANC-1 cells, cells were seeded overnight in 8-well chamber slides at 3x10^4^ cells per well. Cells were then serum starved overnight. On the next day, cells were treated with 1–2 μM AMD3100 or PBS control for 1 h. Cells were washed with PBS-glycine, fixed, permeabilized, and blocked for 1 h at room temperature in 3% bovine serum albumin (BSA) or goat serum (Sigma-Aldrich). Cells were incubated overnight at 4°C with rabbit anti-PD-1 (1:100, Proteintech, 18106-1-AP) and mouse anti-CXCR4 (1:100, Proteintech, 60042-1-Ig) primary antibodies. The following day cells were incubated with goat anti-rabbit Alexa Fluor 488 and goat anti-mouse Alexa Fluor 555 secondary antibodies (1:500, ThermoFisher) for 1 h at room temperature. Secondary antibodies alone were used as antibody controls. VectaSheild with DAPI (Vector Laboratories) was applied to the stained cells and cells were imaged using Nikon Ts2, confocal microscopes, or plain film.

PDOs were embedded in paraffin and processed by the University of Kentucky Markey Cancer Center Biospecimen Procurement and Translational Pathology Shared Resource Facility (BPTP SRF) onto slides at 5 μm thickness. For 3-color IF, slides were deparaffinized with d-Limonene, rehydrated with an alcohol gradient, and rinsed in TBS. For antigen retrieval, HistoZyme (pH 7.2, Sigma) was applied to the PDO sections for 5 min at room temperature, then rinsed in TBS. Slides were blocked in 10% goat serum in TBS for 1 h at room temperature. Slides were incubated overnight at 4°C with primary antibodies diluted as described above in TBS-T. The following day slides were rinsed in TBS-T followed by TBS. Secondary antibodies, described above, were diluted in 1x TBS for 1 h at room temperature. Slides were again rinsed in TBS followed by ddH_2_O. VectaSheild with DAPI (Vector Laboratories) was applied to each PDO section and coverslips were placed. Slides were allowed to dry overnight and imaged using a Nikon confocal microscope.

For 4-color multiplex IF, slides were stained using Ventana Discovery Ultra machine by the University of Kentucky BPTP SRF. Antigen retrieval was performed on-board using CC1 standard, followed by 20-min incubation with mouse anti-α-SMA (Ventana 760–2833) with detection using OmniMap anti-mouse HRP (Ventana 760–4310) and Rhodamine 6G fluorophore kit (Ventana 760–244). Unbound antibody was denatured by heating to 90°C for 4 min and unreacted peroxidase was quenched. Slides were subsequently incubated with mouse anti-PD-1 antibody (Cell Marque 315M-98) for 1 h with detection by OmniMap anti-mouse HRP and FITC kit (Ventana760-232), followed by another round of denaturation and quenching before incubation with mouse anti-CXCR4 (Proteintech, 60042-1-Ig) at 1:100 for 32 min with detection by OmniMap anti-mouse HRP and Cy5 (Ventana 760–238). Slides were counterstained with DAPI (Ventana 760–4196) for 4 min before mounting and coverslip application. Slides were allowed to dry overnight and imaged using a Nikon confocal microscope.

### Co-immunoprecipitation assays

To assess a direct protein interaction between PD-1 and CXCR4 as a potential mechanism for anti-PD-1/anti-CXCR4 therapeutic synergy, co-immunoprecipitation (co-IP) assay was performed. MIAPaCa-2 and PANC-1 cells were exposed to AMD3100 (1 μM) or solvent control and then lysed with Pierce IP lysis buffer (ThermoFisher) containing phosphatase and protease inhibitor cocktails (Sigma-Aldrich). After centrifugation, the protein extracts were subjected to preclearing and immunoprecipitation using Protein A/G-Agarose Plus (Santa Cruz Biotechnology). One ml of precleared cell lysate (1 mg total protein) was incubated overnight at 4°C with rabbit polyclonal PD-1 antibody (10 μg, Proteintech, 18106-1-AP) or rabbit IgG isotype control antibody (Invitrogen) as a pulldown control. Reciprocal IP was performed using mouse anti-human monoclonal CXCR4 antibody (10 μg, Proteintech, 60042-1-Ig). The immunoprecipitated pellets were then washed ×4 and boiled in SDS sample buffer for 5 min at 95°C. After brief centrifugation, the supernatant from each condition was run on an SDS-PAGE gel followed by western blotting to evaluate PD-1 and CXCR4. The SuperSignal^™^ West Pico PLUS Chemiluminescent Substrate Kit (ThermoFisher) was used to visualize the membranes with a UVP ChemiDoc-It2imager or plain film. Blots were quantified using ImageJ software (NIH) and analyses performed using GraphPad software.

### Flow cytometry

Cell surface PD-1 expression was assessed by flow cytometric assays. MIAPaCa-2 and PANC-1 cells were plated on 100 mm^3^ culture dishes and incubated overnight for cell attachment. The next day, cells were serum-starved overnight. On the 3^rd^ day, cells were exposed to AMD3100 (1 μM) or solvent control for 45 min. Cells were then lysed and prepared for flow cytometric analysis of PD-1 expression. Mouse anti-PD-1-PE antibody (20 μL per test, BD Biosciences, 557946) was used to probe for cell surface PD-1 expression. Mouse IgG1-PE antibody was used as isotype antibody control and the assay was performed on an LSR II cell analyzer (BD Sciences). Flowjo v10 software was used for flow data analysis.

### Cell migration assays

To study the potential regulation of PD-1 on CXCR4-mediated migration, MIAPaCa-2 and PANC-1 cells were placed into 24-wells with transwell inserts (8 μm pores; Costar, Corning) to assess the ability of cells to migrate through the membranes [16]. Cells (5x10^4^) in 100 μL serum-free DMEM medium were placed into the upper chamber of each well and 600 μL of DMEM medium containing 1% FBS and AMD3100 (1 μM), pembrolizumab (humanized anti-PD-1 monoclonal antibody; 1 μg/mL), CXCL12 (100 ng/mL), or a combination of these were added to bottom chambers. Since PANC-1 cells have appear to have more rapid migration than MIAPaCa-2 cells [16], cells were incubated for 12 and 24 h, respectively, at 5% CO_2_ and 37°C. After incubation, cells were fixed and permeabilized in ice-cold 100% methanol for 30 min on ice, washed in PBS then stained with crystal violet solution (1% in methanol), and washed with PBS and ddH_2_O. Cells remaining on the top side of each membrane were removed with a cotton swab. The stained transwell membranes were imaged with a Nikon Ts2 microscope and 5 representative images were captured for each membrane. Quantifications were performed using ImageJ and analyses were performed using GraphPad.

### *PD-1* knockdown in MIAPaCa-2 cells

Four constructs of lentiviral short hairpin RNA (shRNA) against human *PD-1* (PDCD1) (NM_005018) or scramble shRNA control (Genecopoeia), with mCherry reporter and puromycin selection genes, were transfected into MiaPaCa-2 cells using the jetPRIME reagent (Polyplus). Cells were selected with puromycin (ThermoFisher) and sorted with Flow Cytometry for further purification. *PD-1* knockdown (KD) efficiency was assessed by western blot and the most efficient PD-1 shRNA constructs were chosen for further study. MIAPaCa-2 *PD-1*-KD cells along with scramble shRNA transfected control cells were used in this study.

### Statistical analysis

Statistical analysis was performed using GraphPad Prism software. One-way ANOVA with post Tukey test was used for multigroup comparisons. Differences were considered significant at *P* < 0.05. Results are expressed as mean ± standard error.

## Results

### PD-1 and CXCR4 are expressed on PDAC cells, tumors, and PDOs

**Fig 1:**
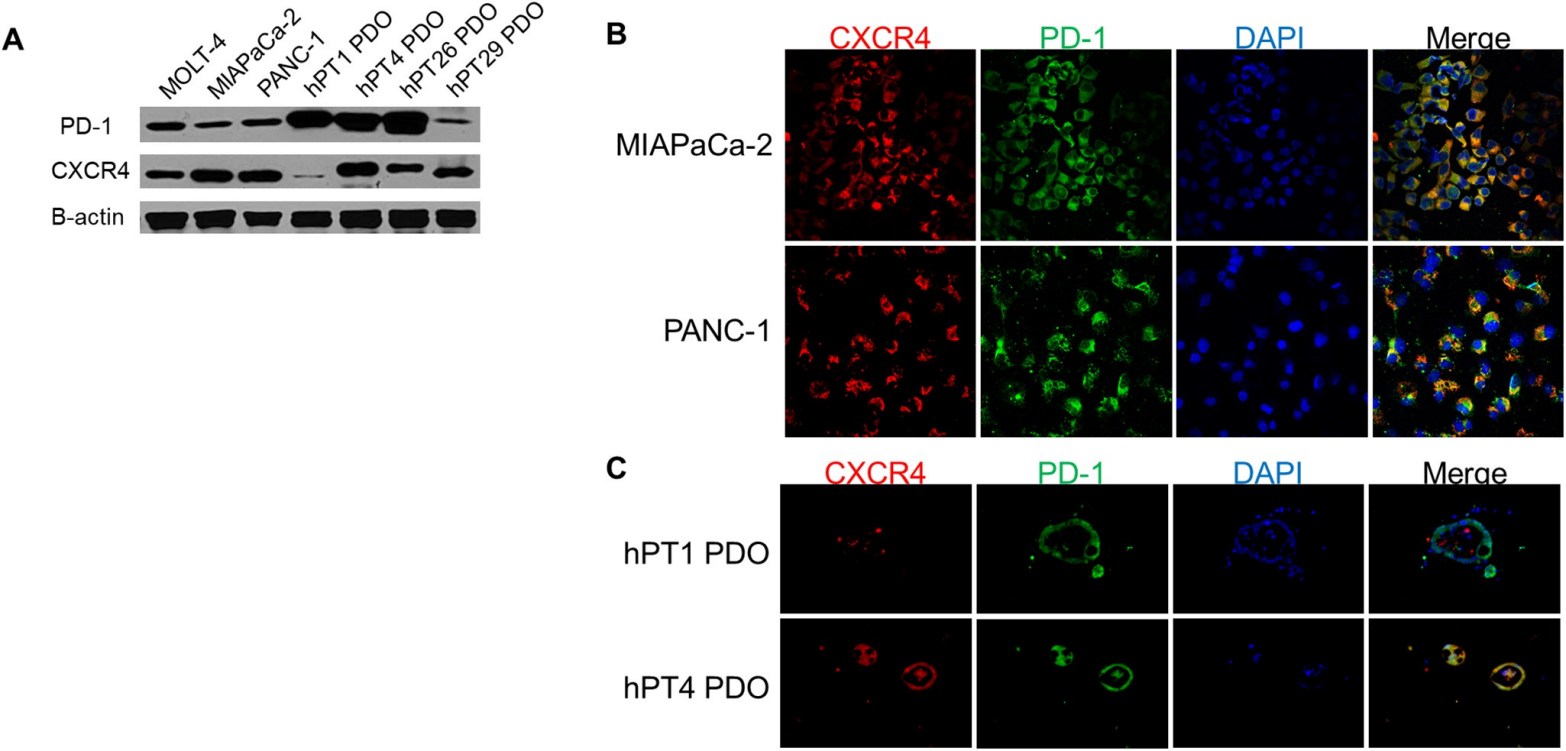
PD-1 and CXCR4 expression in PDAC cells and PDOs. (**A**) PD1 and CXCR4 were detected in PDAC cells and PDOs by western blot. Notably, different cell and PDO lines had unique expression patterns. MOLT-4 was used as a positive control for PD-1 and CXCR4. (**B**) IF staining shows co-expression of PD-1 and CXCR4 in PDAC cells (magnification 40X). (**C**) IF staining shows co-expression of PD-1 and CXCR4 in PDAC PDOs. Consistent with western blot results, hPT1 PDOs had lower expression of CXCR4 compared to hPT4 PDOs (magnification 20X).

**Fig 2:**
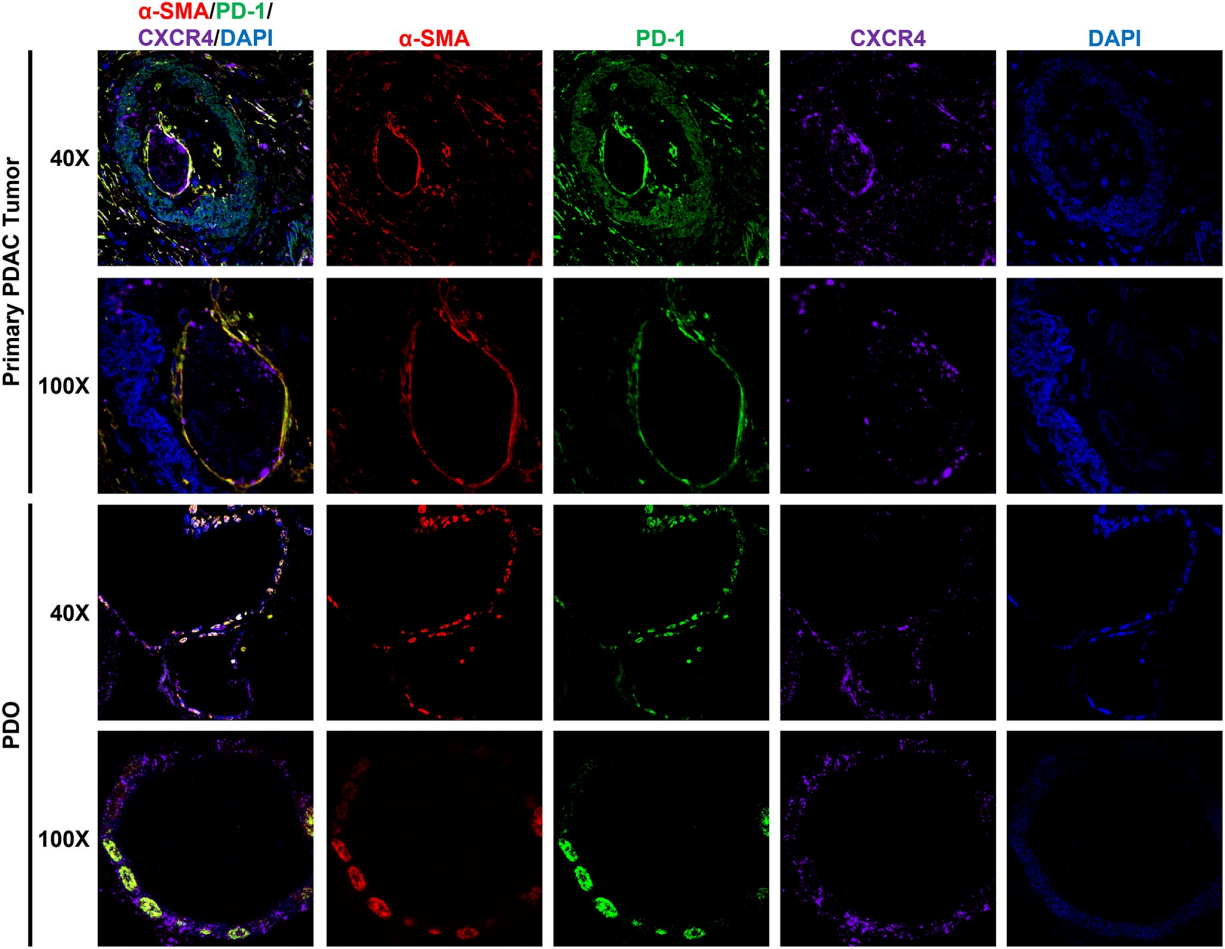
PD-1 and CXCR4 expression in PDAC tumors and PDOs. Operative human primary PDAC specimens and corresponding PDOs for hPT26 were serially sectioned and stained with multiplex IF. Both primary PDAC tissues and PDOs showed co-expression of PD-1 and CXCR4 (**green + violet→ teal**). These regions corresponded to areas consistent with pancreatic duct cells in primary PDAC tumors and PDOs. α-SMA was used as a marker for CAFs, which are known components of the PDAC TME that express PD-1 and CXCR4 (**red + green→ yellow, red + violet→ pink, red + green + violet→ white**). In primary tumors, CAFs with PD-1 and CXCR4 expression were predictably in regions of desmoplasia characteristic of the TME. α-SMA+ CAFs were similarly noted in areas typical of the TME in PDOs, along the periphery of the 3D organoid structures. Altogether, these results show concomitant expression of PD-1 and CXCR4 in human PDAC.

We performed western blot assay to confirm PD-1 and CXCR4 expression in PDAC cells and PDO lines (Fig 1A). The MOLT-4 cell line was used as a positive cell line control for both CXCR4 and PD-1. Then, we performed IF staining on PDAC cell lines (Fig 1B) and PDOs (Fig 1C) to determine if these proteins localized to the same cellular regions. We observed co-expression of PD-1 and CXCR4 in both cancer models. We next sought to determine if co-expression in PDOs was secondary to immune cells or TME cells, such as CAFs, which also express PD-1 and CXCR4 [17–21]. In primary PDAC tissues and corresponding PDOs, PD-1 and CXCR4 co-expression was observed (Fig 2). α-SMA expression, which is more specific to CAFs [22–24] but can also be seen in cancer cells [25–27], was used in combination with surrounding architecture to identify CAFs. We observed that α-SMA negative, PD-1+/CXCR4+ cells in PDAC tissues and PDOs were localized to regions corresponding to ductal and cancer cells.

### PD-1 and CXCR4 interaction in PDAC cells

**Fig 3:**
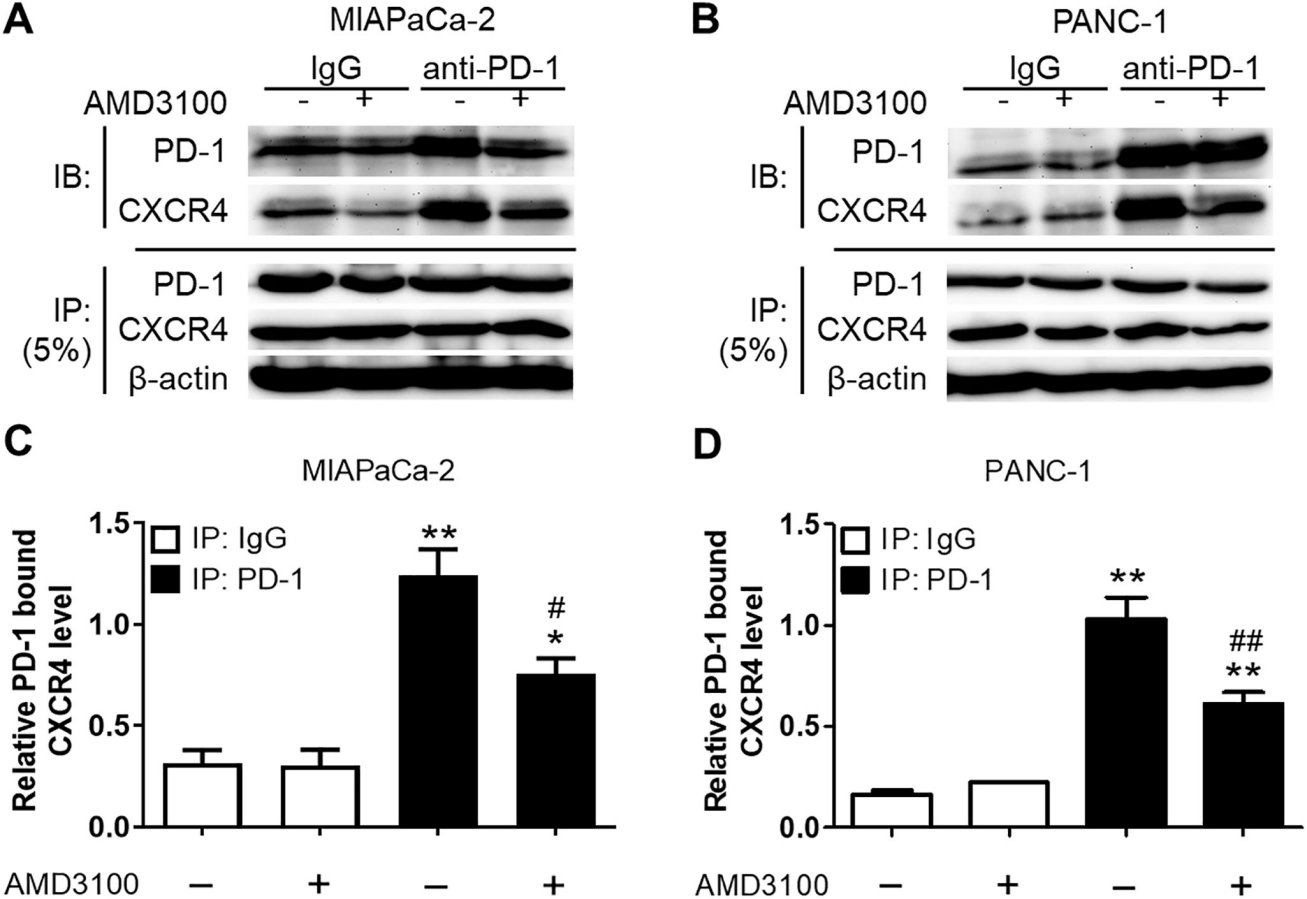
Co-immunoprecipitation studies in MIAPaCa-2 and PANC-1 cell lines. Serum-starved PDAC cells were pre-treated with solvent (-) or AMD3100 (1 μM) (+) for 45 min. Cell lysates were immunoprecipitated with IgG control or anti-PD-1 antibodies. **(A, B)** Immunoprecipitates were immunoblotted (IB) with anti-PD-1 and anti-CXCR4 antibodies, revealing successful pull down of PD-1 and resultant co-IP of CXCR4; 5% of IB lysate (lower panel) was used as input control. Treatment with AMD3100 revealed reduced levels of PD-1-bound CXCR4 in PDAC cells. **(C, D)** Quantification of CXCR4 immunoprecipitated with PD-1 in MIAPaCa-2 (**B**) and PANC-1 (**D**) cells normalized to β-actin as shown in A and C, respectively. *p < 0.05, **p < 0.01 compared with IgG. ^#^*p* < 0.05, ^*##*^*p* < 0.01 compared with solvent control.

Since our prior work and other studies demonstrated potential cross-talk between PD-1 and other oncogenic pathways [6–10], we performed co-IP assays to determine if PD-1 and CXCR4 directly interact in PDAC cells. Immunoblots demonstrated PD-1 and CXCR4 expression following pull-down by anti-PD-1 antibodies, thus revealing a direct protein interaction between PD-1 and CXCR4 in MIAPaCa-2 and PANC-1 cells (Fig 3). We corroborated these findings with reciprocal co-IPs using CXCR4 pull-down (S1 Fig in S1 File). Furthermore, when PDAC cells were exposed to AMD3100 (1 μM), we observed decreased levels of PD-1-bound CXCR4 in both cell lines following pull-down with both antibodies, which further supports the direct interaction between these two proteins. (Fig 3).

**Fig 4:**
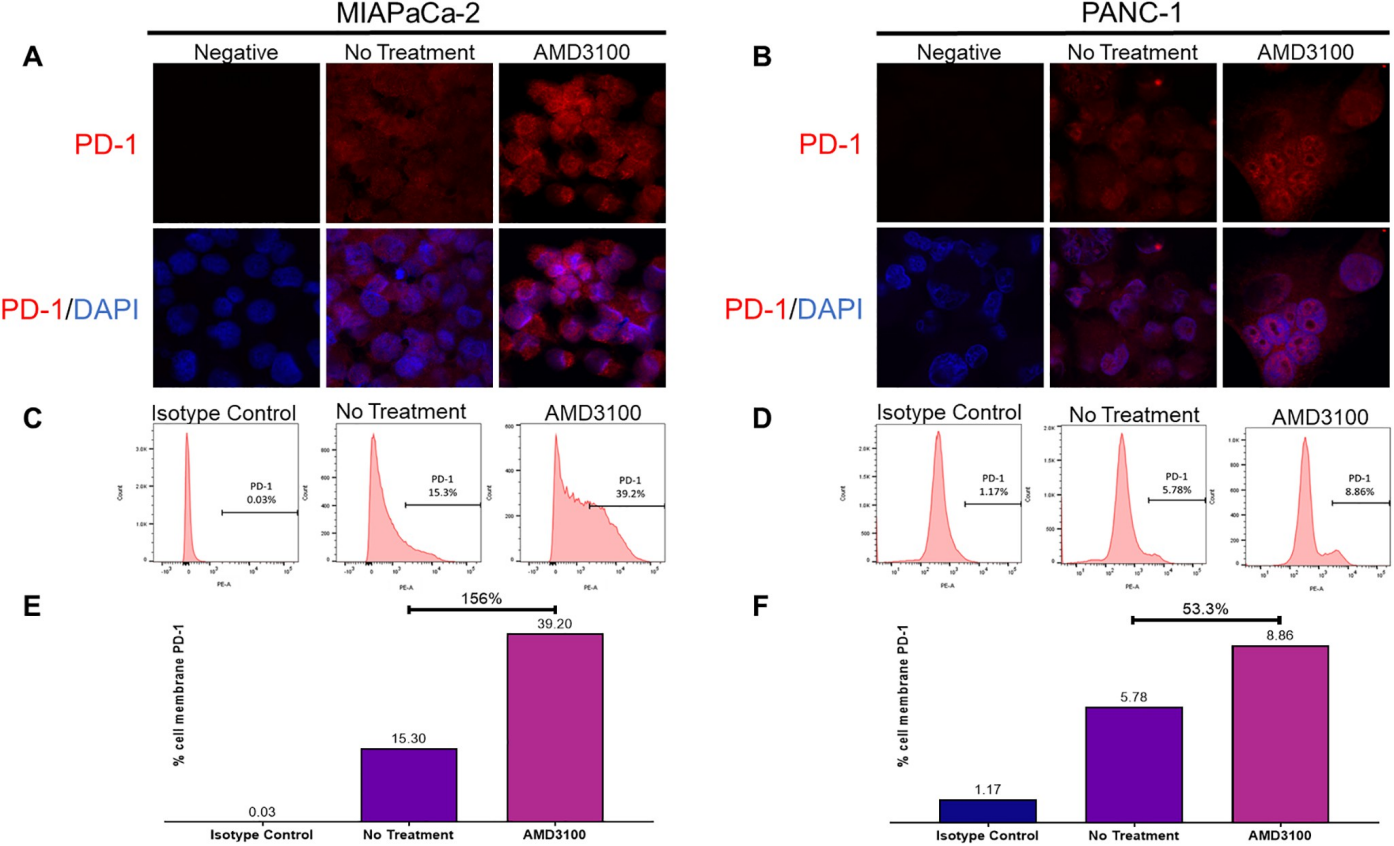
CXCR4 inhibition leads to PD-1 translocation to the cell membrane. (**A, B**) Immunofluorescence analysis of MIAPaCa-2 and PANC-1 PDAC cells. Treatment with AMD3100 resulted in increased PD-1 cell surface expression compared to controls (magnification 100x). (**C, D**) Flow cytometry analysis of PDAC cells. Cells were treated with AMD3100, isotype antibody, or solvent control and then prepared for flow cytometry analysis of PD-1 membrane expression. Treatment with AMD3100 revealed increased surface expression of PD-1 compared to controls. (**E, F**) Quantification of flow cytometry analysis demonstrated 156% and 53.3% increase in cell surface expression of PD-1 after AMD3100 treatment.

We then sought to characterize CXCR4 regulation of PD-1 expression on PDAC cells. IF staining and flow cytometry analysis of PDAC cells following treatment with AMD3100 (or vehicle control) revealed increased cell surface expression of PD-1 in PDAC cells after exposure to AMD3100 (Fig 4), consistent with prior reports [6, 9, 10]. In MIAPaCa-2 cells membrane PD-1 expression increased by 156%, while in PANC-1 cells it increased by 53.3%. These data suggest CXCR4 plays a role in direct or indirect regulation of PD-1 trafficking from the cytoplasm to the cell surface.

### PD-1 inhibition blocks CXCL12-induced PDAC migration

**Fig 5:**
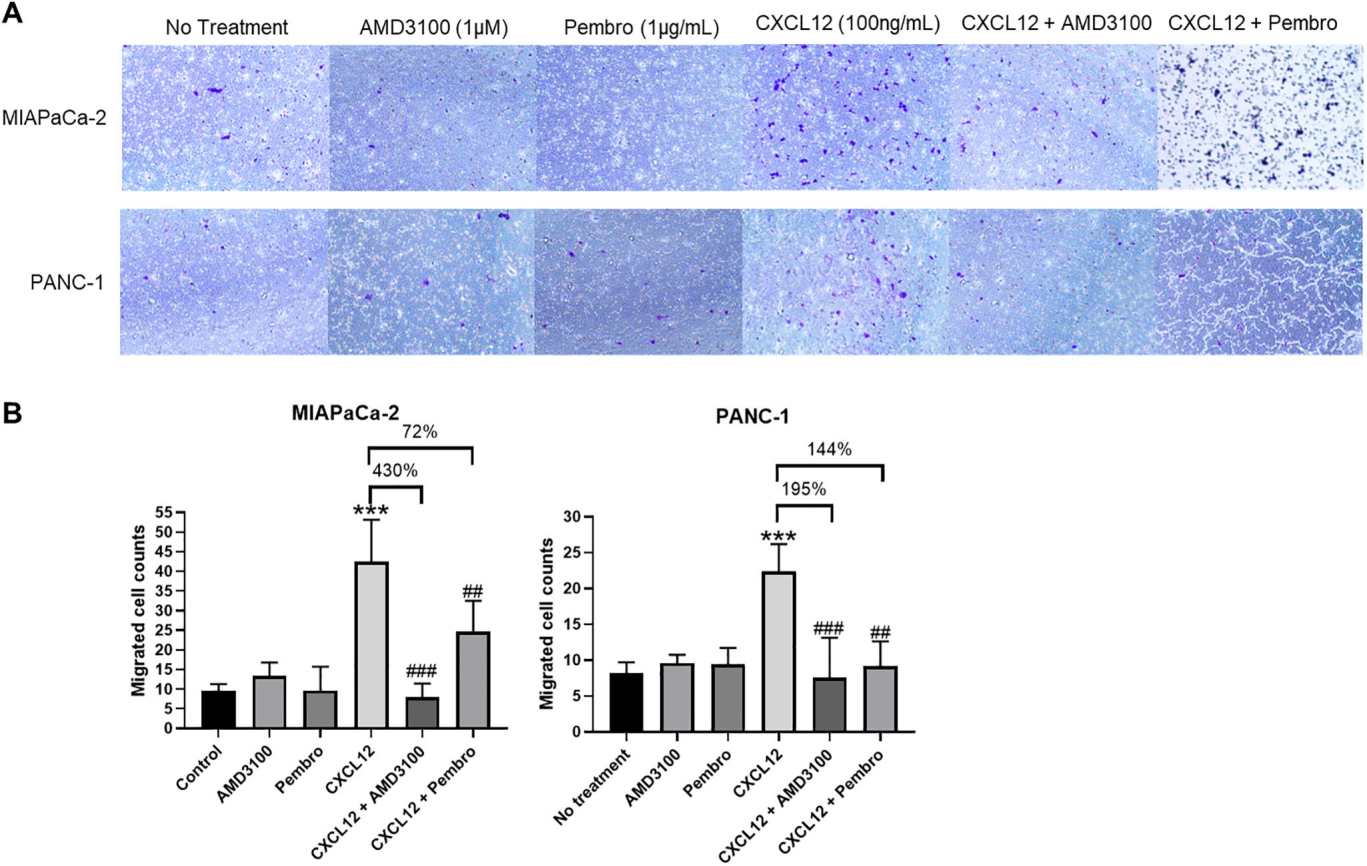
Transwell migration assays of PANC-1 and MIAPaCa-2 cells. (**A**) Treatment with AMD3100 or pembrolizumab alone did not alter cell migration. However, exposure to CXCL12 promoted cell migration in both lines as expected. The addition of AMD3100 or pembrolizumab to CXCL12-treated cells resulted in inhibition of cell migration in both cell lines, demonstrating that inhibition of PD-1 or CXCR4 can block CXCL12-induced migration. All images at 10x magnification. (**B**) Quantification of transwell migration assays revealed that migration was significantly inhibited in CXCL12-treated cells when exposed to AMD3100 and pembrolizumab, demonstrating that combined CXCR4 and PD-1 inhibition abrogated CXCL12-induced migration. ***p<0.001 vs. control; ##p<0.01, ###p<0.001 vs. CXCL12.

We have previously shown that the chemokine CXCL12 enhances PDAC cell migration through CXCR4 activation, and other groups have reported similar results [28–36]. We sought to examine how the PD-1-CXCR4 interaction may regulate CXCL12-induced cell migration in PDAC cells. In brief, PANC-1 and MIAPaCa-2 cells were placed in the upper well of an 8 μm transwell insert in serum-free medium. In the bottom well, AMD3100, pembrolizumab, CXCL12, or a combination of these were added to the medium with 1% serum. Since PANC-1 cells have more rapid migration than MIAPaCa-2 cells [16], cells were incubated for 12 and 24 h, respectively, at 5% CO_2_ and 37°C. Neither CXCR4 nor PD-1 inhibition alone induced cell migration in either line (Fig 5A). As expected, CXCL12 treatment of PDAC cells promoted migration in both lines. However, the addition of AMD3100 or pembrolizumab blocked CXCL12-induced migration in both lines and decreased the percentage of migrated cells by 430% and 72%, respectively, in MIAPaCa-2 cells and by 195% and 144%, respectively, in PANC-1 cells (Fig 5B).

**Fig 6:**
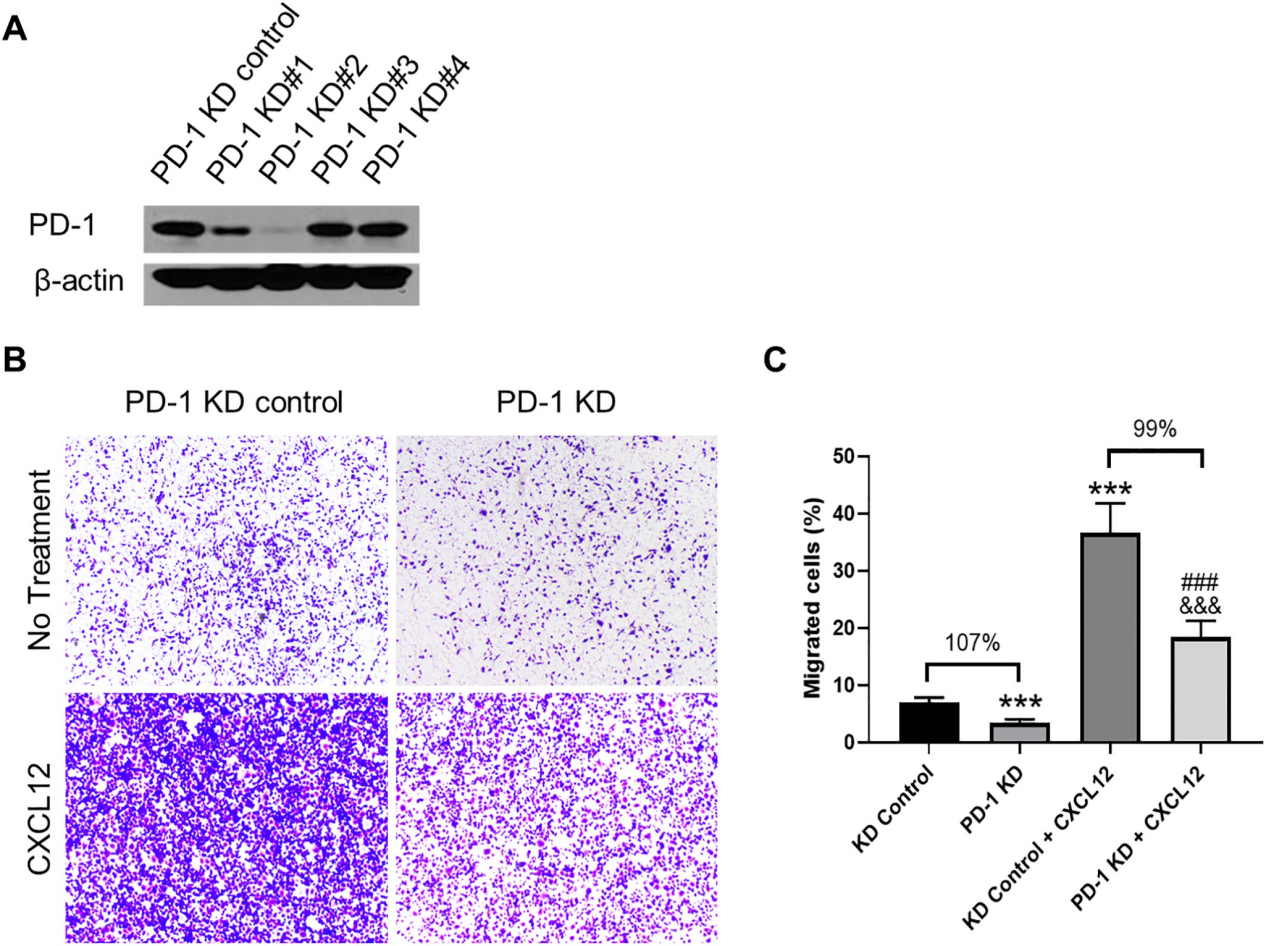
*PD-1* knockdown in MIAPaCa-2 cells attenuates CXCR4 downstream pathways. (**A**) MIAPaCa-2 *PD-1* KD was most successful in decreasing *PD-1* in construct #2. (**B**) *PD-1* KD cells demonstrated decreased migration even when cells were exposed to CXCL12 (100 ng/mL). (**C**) Quantification of transwell migration assays demonstrated a 107% decrease in migration in *PD-1* KD cells compared to KD control. When treated with CXCL12, *PD-1* KD cells had a 99% less migration than KD controls. (*** *p*<0.001 vs. KD control; ### *p* <0.001 vs. *PD-1* KD; &&& *p* <0.001 vs. KD control + CXCL12).

To further investigate the PD-1-CXCR4 interaction and cell migration, we created *PD-1* knockdown (KD) MIAPaCa-2 cells. As shown in Fig 6A, *PD-1* KD construct #2 effectively knocked down PD-1 expression. Transwell migration assays showed diminished migration in *PD-1* KD compared to control KD cells when exposed to CXCL12 stimulation (Fig 6B), further supporting our results in Fig 4 that PD-1 abrogation regulates CXCL12-induced migration. Specifically, PD-1 KD decreased migration by 107% alone, and 99% in the presence of CXCL12 (Fig 6C). Altogether, our results show co-expression and interaction of PD-1 and CXCR4 with combined promotion of PDAC migration.

## Discussion

Although immuno-oncology (IO) drugs have demonstrated clinical efficacy in many cancers [37, 38], they have failed to improve survival in PDAC [1, 2]. More recent clinical trials have taken different approaches, evaluating multi-therapy regimens that include ICIs combined with radiation and chemotherapy [39]. Additionally, select clinical trial regimens are focused on activating the innate immune system to complement ICI therapies [40–42]. CXCR4 has become a target of interest in IO therapies due to its immunogenic effects and overexpression in many cancers. In PDAC, major oncogenic signal transduction cascades are activated by the CXCL12/CXCR4 axis. Due to its ubiquitous downstream effects on cancer survival, CXCR4 serves as a valuable candidate therapeutic target for PDAC.

AMD3100 was the first CXCR4 inhibitor to receive FDA approval for its utility in bone marrow transplantation procedures and is currently approved by the FDA for the treatment of multiple myeloma and non-Hodgkin’s lymphoma [43, 44]. In 2004, AMD3100 was found to suppress CXCL12-induced chemotaxis and inhibit proliferation in CXCR4 (+) PDAC cells [34]. *In vitro*, AMD3100 has sensitized PDAC cells to gemcitabine and immunotherapies [45]. *In vivo*, the safety and dose-limiting toxicity of AMD3100 is currently being evaluated in patients with advanced PDAC (NCT02179970). Additionally, other CXCR4/CXCL12 inhibitors have shown synergy with ICIs *in vitro* and these combination regimens are now being assessed in clinical trials for PDAC and myelofibrosis (NCT04177810, NCT02826486, NCT03168139, and NCT04177810) [9, 10].

Importantly, prior trials assessing combined inhibition of CXCR4 and PD-1 in PDAC aimed to activate the body’s innate immune response. However, no study has assessed the effects of combination therapy directly on PDAC cells. In our previous publication, we reported autonomous expression of PD-1 on PDAC cells, independent of immune cell populations, and that combined targeting of PD-1 and the oncogenic MAPK pathway increased cytotoxicity of PDAC cells [6]. Here, we build on these studies by further characterizing CXCR4 and PD-1 interactions in PDAC cells.

Ours is the first report of a direct interaction between PD-1 and CXCR4 in cancer cells. It is well-established that CXCR4 is overexpressed in over 23 different cancer types, and that PD-1 is expressed not only on immune cells but also in several different cancer cells [6–8, 46–48]. Tumor intrinsic PD-1 has been shown to promote tumor growth in melanoma, ovarian, liver, renal and pancreatic cancers [46]. Thus, expression of CXCR4 and PD-1 in PDAC cells is of great interest since these are traditionally immune specific markers. In fact, our expression patterns are consistent with what is known about G-protein coupled receptors, which undergo internalization after interaction with ligands [49]. Additionally, ligand-induced endocytosis of CXCR4 and its internal sequestration are also well known in leukocytes, stem cells, and tumor cells [50–52].

We theorized a potential direct protein interaction between CXCR4 and PD-1 since therapeutic targeting appears to have synergy and because both localize on the cell membrane and in the cytoplasm. Our co-IP studies support a direct interaction between PD-1 and CXCR4 that was disrupted by AMD3100, indicating direct or indirect mechanism for PD-1 trafficking from the cytoplasm to the cell membrane. This PD-1 and CXCR4 interaction also appears to support PDAC cell migration. Notably, targeting either PD-1 or CXCR4 with pembrolizumab or AMD3100, respectively, both inhibited CXCL12-mediated PDAC cell migration. Although AMD3100 has been shown to have off-target effects and bind to CXCR7, it does so less effectively than with CXCR4 [53]. Furthermore, CXCR7 is required for CXCR4 activation, and thus AMD3100 can also indirectly inhibit CXCR4 by binding to CXCR7 [54, 55]. Together, these conditions reinforce our assertion that our observed results with AMD3100 treatment are due to CXCR4 inhibition.

*PD-1* KD assays further indicated that PDAC cell migration was dependent on both PD-1 and CXCR4 expression. Importantly, our results suggest a potential mechanism for increased cytotoxicity with combination therapy. Exposure to AMD3100 in PDAC cells appears to increase the concentration of PD-1 receptors on PDAC cell membranes potentially increasing its exposure to anti-PD-1 therapies. We are developing immunocompetent PDAC models to examine this question. Overall, our findings provide further mechanistic support for prior *in vitro* and *in vivo* studies demonstrating synergistic anti-tumor effects with combined CXCR4 and PD-1 antagonism.

## Conclusions

To our knowledge, this is the first report to investigate PD-1 and CXCR4 interactions in PDAC models independent of immune components. We previously discovered autonomous expression of PD-1 on PDAC cells and have now built upon our initial studies by evaluating the intracellular interactions between PD-1 and CXCR4, thereby providing a potential mechanism for the clinical efficacy of combination CXCR4 and PD-1 therapy in PDAC. Our studies reveal these interactions are disrupted by drugs targeting CXCR4 or PD-1 in PDAC cells, and that CXCR4 engages PD-1 for activation of downstream effects. In future studies we plan to further elucidate the relationship between PD-1 and CXCR4 in immunocompetent PDAC models, thereby building the groundwork for future clinical trials.

## Supporting information

S1 File (PDF)
