# Supplementary figures and images for: Interaction of immune checkpoint PD-1 and chemokine receptor 4 (CXCR4) promotes a malignant phenotype in pancreatic cancer cells
Source: PLoS One. 2022 Jul 7;17(7):e0270832. doi: 10.1371/journal.pone.0270832 (PMC9262213; doi:10.1371/journal.pone.0270832)

**S1**

**A**

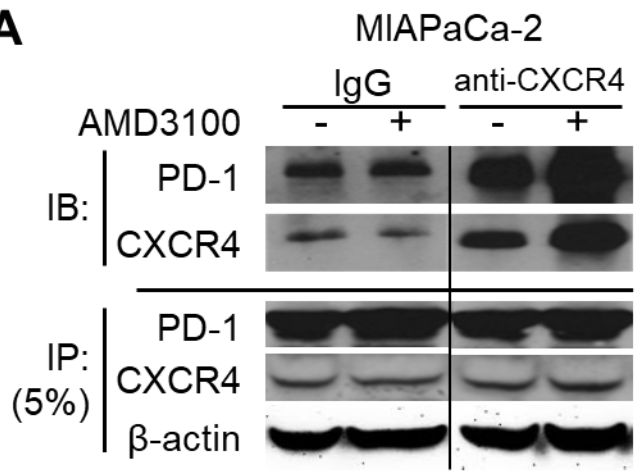

**B**

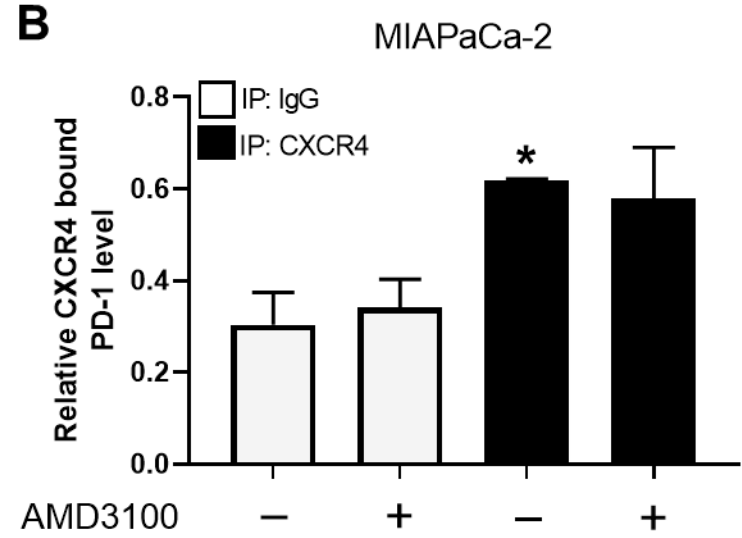

**S2**

MOLT-4      MIAPaCa-2      PANC-1      hPT1      hPT4      hPT26      hPT29

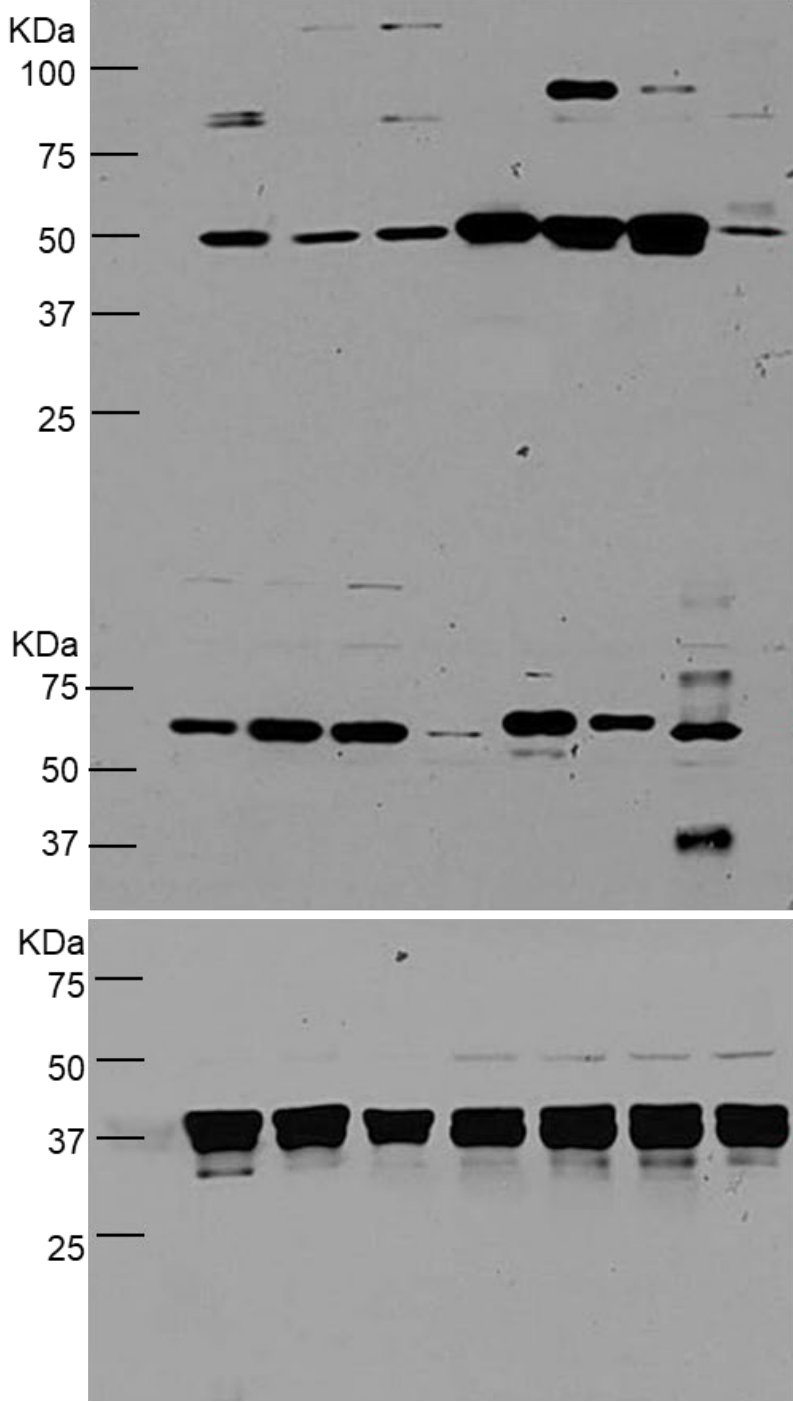

← PD-1 (32, 47-55 kDa)

← CXCR4 (40, 60-70 kDa)

← β-actin (42 kDa)

**B**

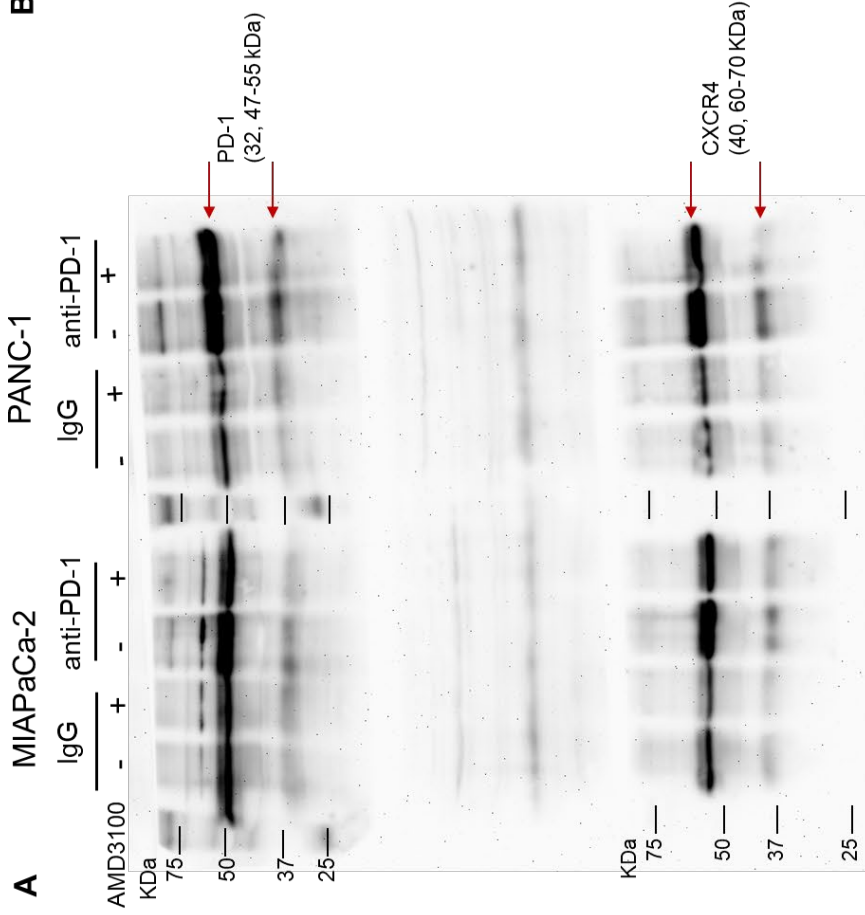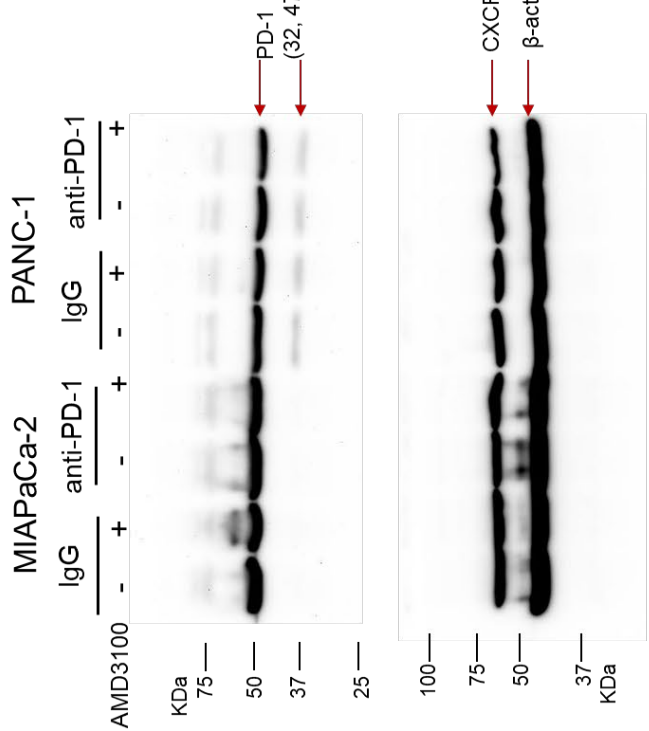

S4

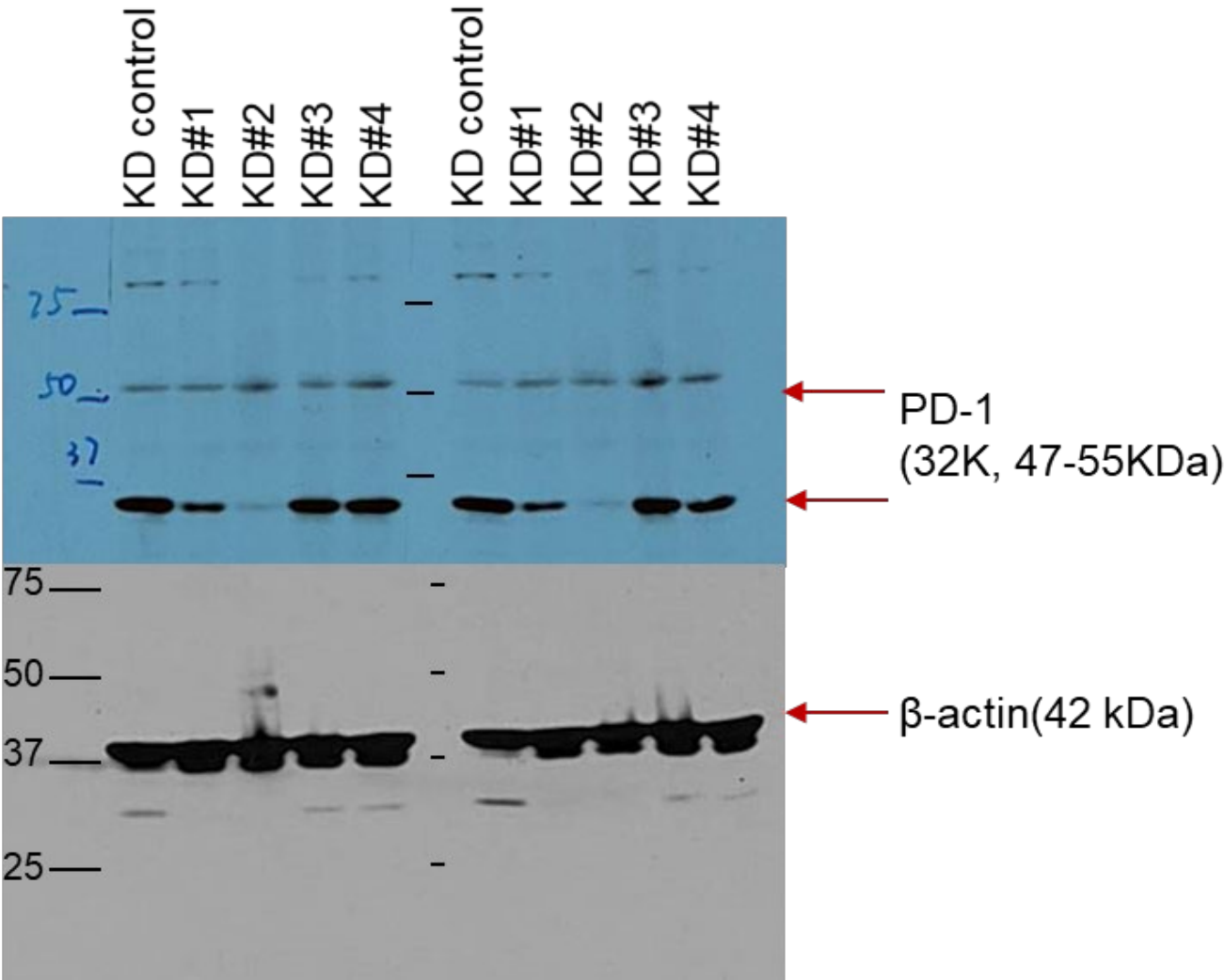

A

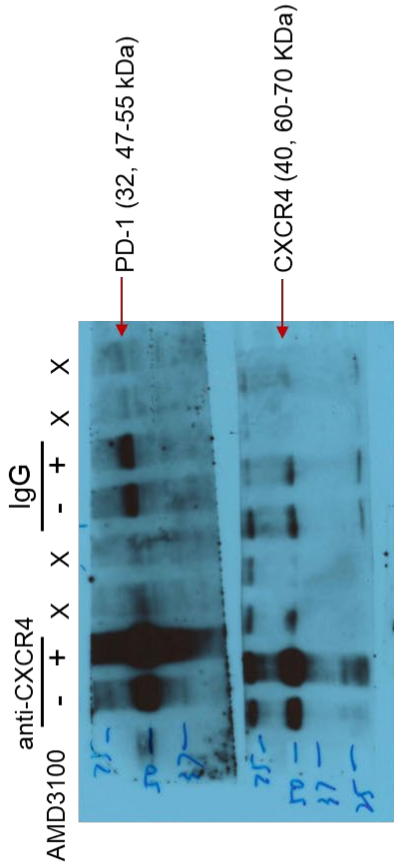

B

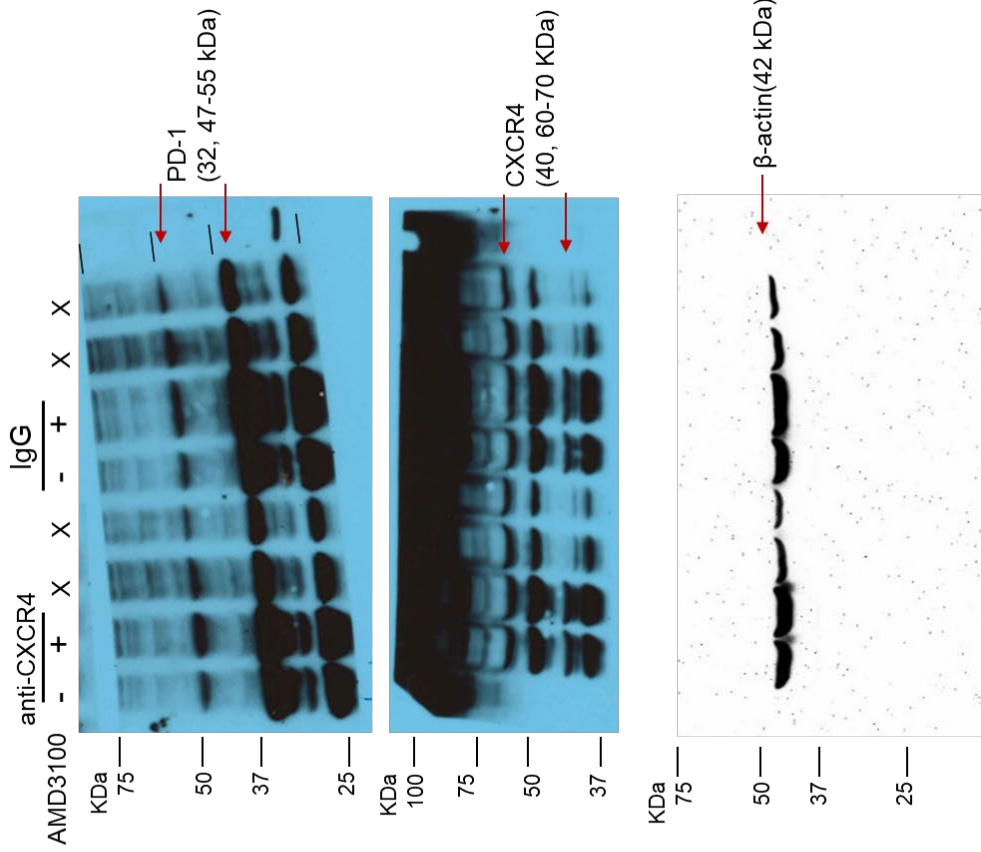

Supplement: S1 File — (PDF) [file pone.0270832.s001.pdf]
